# Predictors of endoscopic transsphenoidal surgery outcome in acromegaly: patient and tumor characteristics evaluated by magnetic resonance imaging

**DOI:** 10.1007/s11102-012-0395-7

**Published:** 2012-04-26

**Authors:** Christa C. van Bunderen, Nadège C. van Varsseveld, Johannes C. Baayen, Wouter R. van Furth, Esther Sanchez Aliaga, Marieke J. Hazewinkel, Charles B. L. M. Majoie, Nicole J. M. Freling, Paul Lips, Eric Fliers, Peter H. Bisschop, Madeleine L. Drent

**Affiliations:** 1 Department of Internal Medicine, Section Endocrinology, VU University Medical Center, ZH 4A62, De Boelelaan 1117, 1081 HV Amsterdam, The Netherlands; 2 Department of Neurosurgery, Endoscopic Skull Base Amsterdam, VU University Medical Center, 1081 HV Amsterdam, The Netherlands; 3 Department of Neurosurgery, Endoscopic Skull Base Amsterdam, Academic Medical Center, 1105 AZ Amsterdam, The Netherlands; 4 Department of Radiology, VU University Medical Center, 1081 HV Amsterdam, The Netherlands; 5 Department of Radiology, Academic Medical Center, 1105 AZ Amsterdam, The Netherlands; 6 Department of Endocrinology and Metabolism, Academic Medical Center, 1105 AZ Amsterdam, The Netherlands

**Keywords:** Acromegaly, GH-secreting pituitary adenoma, MRI, Transsphenoidal surgery, Tumor characteristics

## Abstract

The availability of various first-line treatment modalities for acromegaly and evolving surgical techniques emphasize the need for accurately defined predictors of surgical outcome. We retrospectively analysed the outcome of 30 patients with acromegaly after initial endoscopic transsphenoidal surgery in two university hospitals from 2001 until 2009, and reviewed comparable literature investigating predictive tumor characteristics. Medical records were monitored for patient characteristics. Each pituitary magnetic resonance imaging (MRI) scan was revised independently by two neuroradiologists using a standardised analysis form to record distinctive predefined tumor characteristics. All characteristics were independently analysed as predictors for persistent disease, and a multivariable predictive model was created. Literature from 2000 onwards was searched for studies describing tumor characteristics predictive for surgical outcome. The cohort consisted of 27 macroadenomas with 90 % demonstrating signs of parasellar extension. The surgical cure rate overall was 30 %. Independently, next to male sex and increasing tumor size, infrasellar and parasellar extension based on MRI staging tended to increase the risk of persistent disease. In a multivariable analysis, sex and parasellar extension of the tumor were demonstrated to be the variables allowing for the best fitted predictive model for persistent disease. Earlier studies on preoperative tumor characteristics showed comparable results, although these were based on several different tumor classification systems. This retrospective study demonstrates that accurately defined tumor characteristics based on imaging, especially for cavernous sinus invasion, can be helpful in predicting surgical outcome. Comparative studies on different treatment modalities are essential for clinical practice within the scope of re-evaluation of the role of surgery in GH-secreting adenomas.

## Introduction

Growth hormone (GH)-secreting pituitary adenomas lead to the clinical picture of acromegaly. Symptoms and signs of GH hypersecretion can range from subtle acral overgrowth or soft tissue swelling to diabetes and cardiac failure. Visual field defects and headache, accompanying expanding tumor may be part of this presentation [1, 2]. Acromegaly is considered a serious disorder, which—if left untreated—is associated with an increased morbidity and mortality rate [3]. Treatment of choice is transsphenoidal surgery (TSS) for microadenomas, for non-invasive macroadenomas, and for adenomas causing compression symptoms. The first pituitary surgery for acromegaly was performed in 1893 in England [4], while the first transsphenoidal successes were obtained in 1907 by, among others, Harvey Cushing in the US [5]. Nowadays, large series have shown that TSS is an effective therapy with acceptable low complication and mortality rates [6]. One of the largest cohorts demonstrated remission rates of non-invasive adenomas of 72.2 %, dropping to 21.6 % for invasive adenomas [7]. In the UK it was shown that surgical outcome varied widely between centres (20–60 %) and that surgical experience was an important determinant [8]. However, the influence of tumor characteristics such as tumor size on surgical outcome is still unclear. Macroadenomas with cavernous sinus invasion—aside from factors such as young age and high pre-treatment levels of GH—are unlikely to be controlled by surgery alone [9]. Medical therapy before surgery was initially suggested for patients with severe cardiopulmonary complications of acromegaly. More recently, first-line drug treatment—with or without primary surgical debulking—was also suggested for patients with a low probability of surgical cure [10, 11]. Together with improvements in radiotherapeutic techniques these developments have led to the idea of refraining from surgery in the cavernous sinus. Both developments stress the need for accurate radiological predictors of surgical outcome. A classification system for defining tumor size and invasiveness is needed as exemplified by the recently updated guidelines for acromegaly management [12]. Magnetic resonance imaging (MRI) is now the reference standard for analysing pituitary adenomas, providing invaluable information about tumor size and extension [13]. Crossing of the intercarotid lines and the percentage of intracavernous internal carotid artery (ICA) encasement by the tumor are proven feasible criteria for assessing cavernous sinus invasion [14, 15]. Some tumor staging classification systems have been proposed [16–18], but these are not widely used. Predicting surgical outcome on the basis of preoperative pituitary MRI has not been fully worked out in acromegaly.

The aim of this retrospective study was to review surgical outcome in a group of acromegaly patients who underwent pure endoscopic surgery in two university hospitals in Amsterdam, the Netherlands, from 2001 until 2009. Patient and tumor characteristics—evaluated by blinded, standardised revision of pituitary MRI—were investigated for predictive value on initial surgical outcome. Literature was searched for studies investigating predictors of surgical outcome based on pituitary imaging from 2000 onwards.

## Subjects and methods

### Subjects

We conducted a retrospective review of medical records of 30 adult patients with acromegaly who underwent pituitary surgery and were seen for follow-up in two university hospitals (VU University Medical Center (VUmc) n = 21, and Academic Medical Center (AMC) n = 9) in Amsterdam, the Netherlands. For data on long-term outcome two patients were lost to follow-up because treatment was continued outside our university hospitals. Patients underwent surgery between March 2001 and December 2009 by a total of three different neurosurgeons over time.

### Clinical investigations

Medical records, including laboratory investigations and surgical reports, were monitored for patient characteristics. Different symptoms of GH hypersecretion or tumor expansion were registered. Date of diagnosis of acromegaly was the date of demonstration of elevated levels of insulin-like growth factor-I (IGF-I) adjusted for age and sex, or GH levels above reference range, both accompanied by clinical symptoms of acromegaly. The diagnosis was confirmed in all cases by an oral glucose tolerance test (OGTT), and a nadir GH during an OGTT of <3 mU/L (1 μg/L) excluded acromegaly. Follow-up started on the date of the first surgery. In all cases efforts were made to remove as much tumor tissue as possible and to preserve the function of the surrounding normal pituitary gland. Surgical complications were recorded. Endoscopic surgery was routinely performed as first-line treatment, sometimes preceded by medical therapy. Preoperative treatment with GH-lowering drugs, dopamine agonists or somatostatin analogues, was registered. Hypopituitarism—both before and after surgery—was evaluated using laboratory investigations, stimulatory tests or basal plasma hormone levels together with matching substitution therapy prescribed by the attending endocrinologist, obtained from the medical records.

Remission after surgery was retrospectively based on the opinion of the attending endocrinologists. They had based their judgement on the consensus at that time, aiming at normal IGF-I levels adjusted for age and sex and the absence of active clinical symptoms. An OGTT was occasionally done. Postoperative remission was verified if the patients were still having normal IGF-I or random GH levels at later follow-up, no clinical symptoms of acromegaly and no new therapy was initiated. Patients receiving GH-lowering drugs after surgery were not classified as being in remission at long-term evaluation of outcome, despite reaching normal IGF-I levels. A recurrence was defined as an initial remission followed by a considerable rise in GH or IGF-I levels, recurrent symptoms or newly introduced therapy.

### Endocrine evaluation

GH and IGF-I levels were determined in two different endocrine laboratories. Preoperative GH and IGF-I levels were monitored as a basal measurement without the influence of GH-lowering drugs. The assays used for hormone measurements changed over the long time period of the study. At the VUmc GH was assayed by an immunometric assay (Sorin Biomedica, Italy) until 2002, an immunoluminometric assay (ILMA) (Advantage, Nichols Institute Diagnostics, USA) until 2006, followed by an ILMA (Immulite 2500, DPC/Siemens Diagnostics, LA, USA) onwards. At the AMC GH was assayed by ILMA (Nichols Institute Diagnostics) until 2007, and by immunofluorescence assay (IFMA) (DELFIA, PerkinElmer Inc., UK) onwards. The GH assays were calibrated against the WHO second International Standard 98/574. At the VUmc, serum IGF-I was determined by IRMA (DSL, Webster, TX, USA) until 2001, and by ILMA (Liaison and Advantage, Nichols Institute Diagnostics,) until 2006, followed by chemiluminescence (Immulite 2500, DPC/Siemens Diagnostics) onwards. At the AMC, for IGF-I determination IRMA (DSL) was used until 2004 and ILMA (Nichols Institute Diagnostics) until 2006. From 2006 chemiluminescence (Immulite 2000, DPC/Siemens Diagnostics) was used to determine IGF-I. The IGF-1 assays were standardized to the WHO International Reference Reagent 87/518. When necessary, conversion factors based on method comparisons performed by both laboratories were applied to all IGF and GH values to make these comparable. The results of IGF-I concentration are expressed as the percentage of the upper limit of current normal range (%ULN) for age and sex [19].

### Neuroradiologic evaluation

Of the 30 patients included in the study all had retrievable preoperative imaging of the pituitary, being an MRI scan with thin section T1-weighted spin echo sequences in sagittal and coronal planes, usually with intravenous contrast (gadolinium). Two experienced neuroradiologists, being unaware of surgical outcome, evaluated each MRI scan independently. All MRI scans were revised twice and the results are presented as means. A systematic, standardised pituitary MRI analysis form was constructed using criteria formulated by Bourdelot et al. [18] with a few modifications. The percentage of intracavernous ICA encasement and the length of this encasement along the artery in the cavernous sinus—measured in millimetres—were added as criteria.

Medline was searched using the terms “acromegaly” and “surgery”. Studies from 2000 onwards were selected on title and abstract for those investigating predictors of surgical outcome in GH-secreting adenomas, and based on the content which in particular described the classification method for tumor characteristics.

### Statistical analyses

Categorical data are expressed as number (percentage) and continuous data as median (range) due to skewed distributions. Analyses included Student’s *t* tests, Chi-square tests or non-parametric tests when appropriate. Logistic regression was used to analyse predictors of initial surgical outcome. The continuous variables were tested for linearity and when indicated dummy variables were used. In the univariate analyses odds ratio’s (OR) and 95 % confidence intervals (CI) were determined for all patient and tumor characteristics. For the univariate analysis a significance level of 0.05 was used. Multivariable analysis was performed using backward logistic regression. For this analysis a more liberal *p* value of <0.20 was used with respect to variable selection because a *p* value of 0.05 can lead to selection bias and optimism as a result of overfitting, meaning that the model is too closely adapted to the data [20]. Subsequently, due to the relatively small number of patients a more liberal *p* value is desirable. The quality of the model was tested by the Hosmer–Lemeshow test for goodness-of-fit, by calculating the c-statistic (area under the curve) for discriminative ability and R square for the explained variance. The statistical analyses were performed by the statistical software package SPSS version 15.0 for Windows (SPSS Inc., Chicago, IL, USA).

## Results

### Patient characteristics

In 30 patients (median age 43.6 (23.9–65.3), 40 % women) data on follow-up was available. Median follow-up was 1.1 years (0.3–7.6). Table 1 shows the patient characteristics at baseline. The cohort included 27 macroadenomas (tumor size >10 mm) and 3 microadenomas. There was a considerable range in time between diagnosis and surgery and preoperative GH and IGF-I concentrations. Of the patients 37 % received GH-lowering drugs prior to surgery, either somatostatin analogues (91 %) or dopamine agonists (9 %). Hypopituitarism was described in 30 % of patients preoperatively, with gonadal deficiency being most prevalent. Of symptoms related to tumor expansion, headache was present in 41 % of patients. Visual field defects were reported in 7 patients (23 %), all having a macroadenoma at initial evaluation.

**Table 1:** Clinical patient characteristics prior to surgery

	n (%)	Median	Range
Age (years)		43.6	23.9–62.3
Men	18 (60)		
Time between diagnosis and surgery (years)		0.4	0–4.5
Preoperative basal GH (mU/L)		27	7.6–762.0
Preoperative IGF-I (%ULN)		316	184–539
Tumor size > 10 mm	27 (90)		
Received prior GH-lowering medication	11 (37)		
Hypopituitarism (≥1 axis)	9 (30)		
Hypogonadism	7 (23)		
Hypocortisolism	5 (17)		
Hypothyroidism	4 (13)		
Diabetes insipidus	0 (0)		
Symptoms at presentation			
Acral enlargement	22 (76)		
Snoring	13 (45)		
Macroglossia	13 (45)		
Headache	12 (41)		
Increased perspiration	10 (35)		
Facial changes	9 (31)		
Visual field defect	7 (23)		
Other visual disturbance	4 (13)		

*ULN* upper limit of normal range

### Tumor characteristics

Two pair of neuroradiologists revised 30 pituitary MRI scans on the standardised analysis form, assessing tumor size and extension (suprasellar, infrasellar and parasellar). Additionally, encasement of the ICA was determined, and if this exceeded 25 %, the length along the artery was measured. The combined results are presented in Table 2. Forty-seven percent of revised adenomas were larger than 20 mm. Suprasellar extension was present in 77 % of the adenomas, extension in the sphenoidal sinus (infrasellar) in 50 % and suspected extension into the cavernous sinus (parasellar) in 90 % of cases, including 17 % massive extension. Encasement of the ICA was measured in percentages and divided into four groups, <25 %, 25–50 %, 50–75 %, >75 %. Twenty patients (67 %) demonstrated encasement over 25 %. The encasement was more than 75 % in 10 % of the patients, with a median length over the artery of 15 mm.

**Table 2:**
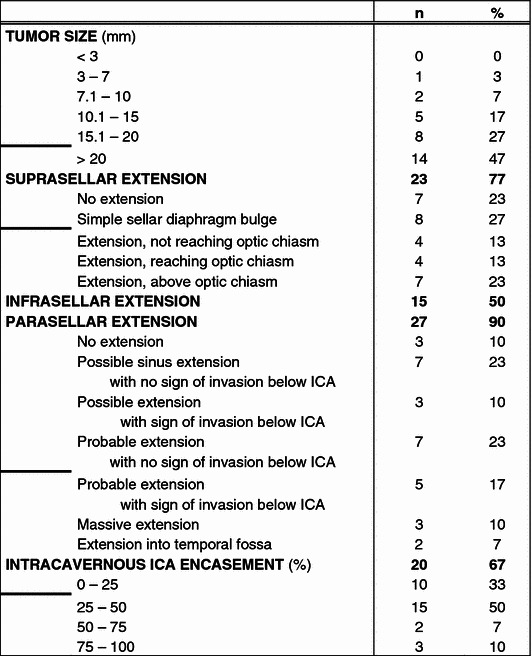
Results of standardised classification of GH-secreting adenoma on MRI scan

*ICA* internal carotid artery

Bold horizontal lines indicates most distinctive cut-off values per characteristic for regression analysis

### Surgical outcome

The initial remission rate of the total cohort was 30 %. Of the 21 patients with persistent disease after the first surgery, 16 received GH-lowering medical treatment. Four patients underwent a second TSS and one received radiotherapy, after which one patient achieved biochemical remission. Long-term remission after multiple therapies was reached in 32 %, excluding patients who achieved biochemical remission with GH-lowering drugs. Of the 9 patients with initial remission, one (11 %) suffered recurrence of the GH-secreting adenoma. During follow-up, no patient died. In 80 % of cases surgical complications were not reported. Leakage of cerebral spinal fluid was the most frequent complication (13 %) and could be managed by lumbar drainage in two cases, and resolved without intervention in the remaining two patients. Hypopituitarism was present in 50 % of patients after surgery, compared to 30 % before surgery. Gonadal and adrenal insufficiently were the most prevalent pituitary insufficiencies. Persistent diabetes insipidus occurred in two cases. Of the seven patients complaining of visual field defects, four patients reported significant improvement postoperatively. In one of these cases this information was lacking.

### Predictors of initial surgical outcome

When only investigating patient characteristics, being a male patient significantly increased the odds for persistent disease after initial pituitary surgery. Preoperative hypopituitarism and short and long time between diagnosis and surgery (compared to intermediate) showed a *p* value of 0.17. Receiving GH-lowering medication prior to surgery tended to lower the odds for persistent disease (OR 0.32, 95 % CI 0.06–1.62; *p* = 0.17). Because medical pre-treatment was not randomised in this study, prognostic factors were compared between treated and untreated patients (data not shown). There were no significant differences. Tumor characteristics based on the standardised revision of pituitary MRI were stratified by most distinctive cut-off value (indicated in Table 2), resulting in: tumor size >20 versus <20 mm, suprasellar extension without optic chiasm involvement or more versus simple sellar diaphragm bulge or less, and probable parasellar extension with sign of invasion below ICA or more versus probable parasellar extension with no sign of invasion below ICA or less. The encasement of the ICA was studied as >25 % and more than 6 mm in length versus <25 % or less than 6 mm in length. Univariate analysis of tumor characteristics showed tumor size, infrasellar extension and parasellar extension to tend to be independent predictors of persistent disease, reaching borderline significance levels. When a multivariable predictive model was made for both patient and tumor characteristics, sex and parasellar extension were included to make the best fitted model (Hosmer–Lemeshow test *p* = 0.84), with an area under the curve of 0.83 (95 % CI 0.68–0.98) and an explained variance of 42 % (Table 3).

**Table 3:** Univariate (A) and multivariable (B) logistic regression analysis of patient and radiologic tumor characteristics as predictors of persistent disease

	Events/n	OR	95 % CI	*p* value
A				
*Patient characteristics*				
Age (years)				
Age < 40 years	8/11	1.91	0.33–11.01	0.47
Age 40–55 years	7/12	1.00		
Age > 55 years	6/7	4.29	0.39–47.63	0.24
Men	16/18	11.20	1.74–72.30	0.01
Preoperative GH (>27 mU/L)	9/12	1.71	0.29–10.30	0.56
Preoperative IGF-1 (%ULN)		1.00	1.00–1.01	0.32
Time between diagnosis and surgery (years)				
First tertile (mean: 0.04)	8/10	4.00	0.55–29.10	0.17
Second tertile (mean: 0.30)	5/10	1.00		
Third tertile (mean: 1.40)	8/10	4.00	0.55–29.10	0.17
Received prior GH-lowering medication	6/11	0.32	0.06–1.62	0.17
Preoperative hypopituitarism (≥1 axis)	8/9	4.92	0.52–47.07	0.17
*Radiologic tumor characteristics*				
Tumor size	12/14	4.67	0.78–28.05	0.09
Suprasellar extension	11/15	1.38	0.29–6.60	0.69
Infrasellar extension	13/15	5.09	0.94–34.46	0.06
Parasellar extension	9/10	6.00	0.63–57.00	0.12
Intracavernous ICA encasement	10/12	3.18	0.53–19.05	0.21
B				
Men	16/18	11.47	1.61–81.91	0.02
Parasellar extension	9/10	6.24	0.53–74.24	0.15

*ULN* upper limit of normal range, *ICA* internal carotid artery

## Discussion

In this retrospective study reviewing surgical outcome in a group of 30 acromegaly patients treated in two university hospitals in Amsterdam, the initial cure rate was 30 %, regardless of tumor size. Tumor characteristics were evaluated twice by standardised revision of pituitary MRI scans by experienced neuroradiologists. Male sex and cavernous sinus invasion of the tumor were predictive characteristics for persistent disease in a multivariable regression analysis.

Surgical cure rates for acromegaly patients have been studied in several cohorts with different numbers of cases, and using different definitions of cure, but in general ranges between 40 and 80 %. The cure rate for microadenomas is much higher (up to 90 %) than for macroadenomas (25–70 %), especially invasive macroadenomas [7, 21, 22]. The relative low cure rate found in our study could be explained by the high percentage of macroadenomas, including 90 % of adenomas with signs of parasellar extension. During the 9 years reviewed in our study, the pituitary surgeries were performed by three different neurosurgeons. This is not an ideal study situation as surgical experience has been associated with better outcome in several studies [8, 23]. Though, we believe this situation did represent clinical practice at time. The need for specialist pituitary surgeons is growing. The consequences of residual GH hypersecretion are not trivial. Further reduction of GH levels is required to eliminate the excess mortality and morbidity. In our cohort almost all postsurgical increased GH levels were eventually controlled with GH-lowering medication.

Next to sex, tumor characteristics were helpful predictors of initial surgical outcome, i.e. persistent disease. We searched the literature for earlier studies specifically investigating predictive tumor characteristics for surgical outcome based on pituitary imaging (Table 4). Only four studies, including present study, investigated purely endoscopic TSS. Characteristics leading to persistent disease after pituitary surgery were an invasive, extrasellar macroadenoma. When trying to specify which radiologic measurements are distinctive, the results were not conclusive. Tumor size ranging from 10 to 20 mm, extension including involvement of the third ventricle and the cavernous sinus, measured in many different ways, were independent predictors. Multivariable analyses demonstrated only tumor size over 15 mm, infrasellar and parasellar extension, and dural invasion to be determinants of persistent disease. Most studies concerned only MRI, but some also included CT scans. The results of the present study are best comparable to the study conducted by Bourdelot et al. [18] because the same radiologic staging system was used, with only a few modifications. We added an extra grade in suprasellar extension with the intention to create a more detailed cut-off point. The measurement of percentage of encasement of the ICA was added because literature demonstrated this to be one of the most valuable criteria for cavernous sinus invasion [24]. In our study tumor size below or above 20 mm was most distinctive to predict surgical outcome while in the study of Bourdelot et al., this threshold was at 15 mm. In both studies infrasellar extension into the sphenoidal sinus was an independent and important factor, as was parasellar extension. Bourdelot et al. studied a relatively larger number of MRI scans but they were only revised once. To preclude inter-observer variability all MRI scans in our study were revised twice by two pairs of experienced neuroradiologists. Since Bourdelot et al. included patients from 1988 onwards; probably most of them underwent microscopic surgery. Present study only included endoscopic surgery, the surgical technique used nowadays.

**Table 4:** Overview of univariate and multivariable analysis of predictive tumor characteristics of surgical cure (A) and persistent disease (B) published from 2000 onwards

References	No. patients	Imaging method	Method of tumor classification	Predictors of cure (univariate analysis)	Predictors of cure (multivariable analysis)
A					
Biermasz et al. [31]	59	NR	Size: according to Hardy and Wilson [16, 17]	None	
			Extension: according to Hardy and Wilson [16, 17]		
			Invasion: based on surgery		
De et al. [32]	90	MRI	Size: micro/macro (>10 mm)	Size: micro	
		+ CT	Extension: intrasellar/extrasellar	Extension: intrasellar	
			Invasion: NR		
Minniti et al. [33]	92	MRI	Size: micro/macro (>10 mm)	Size: micro	Size: micro
			Extension: according to Wilson [17]		
			Invasion: based on surgery		
Nomikos et al. [7]	688	MRI	Size: micro/macro (>10 mm)/giant (>40 mm)	Size: micro	
		+ CT	Extension: NR		
			Invasion: based on surgery (infiltration)	Invasion: non-invasive	
Attanasio et al. [34]	96	MRI	Size: micro/macro (>10 mm)	None	None
			Extension: intrasellar/extrasellar		
			Invasion: expanding outside pituitary fossa		
Jane Jr et al. [35]^a^	60	MRI	Size: micro/meso (>10 mm)/macro (>20 mm)	Size: negative correlation	
			Extension: according to Knosp [15]	Extension: grade 0–II	Extension: grade 0–II
			Invasion: grade III/IV		

References	No. patients	Imaging method	Method of tumor classification	Predictors of persistent disease (univariate analysis)	Predictors of persistent disease (multivariable analysis)
B					
Kreutzer et al. [36]	57	NR	Size: micro/macro (>10 mm)		
			Extension: NR	Extension: extrasellar	
			Invasion: based on surgery + pathology	Invasion: dural invasion	Invasion: dural invasion
Beauregard et al. [37]	103	NR	Size: micro/macro (>10 mm)		
			Extension: based on Hardy and Wilson [16, 17] (4 grades)		
			Invasion: grade III/IV	Invasion: grade III/IV	Invasion: grade III/IV
Minniti et al. [33]	92	MRI	Size: micro/macro (>10 mm)		
			Extension: according to Wilson [17]	Extension: stage B/C and E	Extension: stage E
			Invasion: based on surgery	Invasion: dural invasion	
Bourdelot et al. [18]	83	MRI	Size: 7 grades	Size: > 15 mm	Size: > 15 mm
			Extension: SSE (4 grades), ISE, ICE (7 grades)	Extension: SSE, ISE, ICE	
			Invasion: reaching optic chiasm	Invasion: invasion	
Kim et al. [38]	42	MRI	Size: micro/macro (>10 mm)	Size: macro	
		+ CT	Extension: according to Knosp [15]		
			Invasion: grade III/IV	Invasion: grade III/IV	
Gondim et al. [28]^a^	67	MRI	Size: micro/macro (>10 mm)		
			Extension: according to Hardy [16]	Extension: class D, III/IV	
			Invasion: NR		
Campbell et al. [39]^a^	26	MRI	Size: micro/macro (>10 mm) and volume	Size: tumor volume	
			Extension: according to Hardy [16]	Extension: class III/IV	
			Invasion: according to Knosp [15]	Invasion: grade III/IV	
Present series, 2012^a^	30	MRI	Size: 7 grades		
			Extension: SSE (5 grades), ISE, ICE (7 grades)		Extension: ICE
			Invasion: encasement ICA (4 grades)		

*NR* not reported

^a^ Pure endoscopic transsphenoidal surgery

Different tumor staging classification systems have been proposed. The classification used in the studies reviewed (Table 4) varied widely: some only investigated tumor size based on diameter, others included measures of extension and invasion of the tumor. The tumor classification systems of Hardy [16] and Wilson [17] were most often used. Two studies used Knosp classification [15] to grade invasion of the cavernous sinus based on crossing of medial or lateral intercarotid lines. Another definition of invasion was the judgement of infiltration of tumor tissue in anatomical confines during surgery or by microscopic evaluation of the removed tumor. A practical radiologic classification system for defining invasiveness is desirable, especially in a time when more alternative treatment options are becoming available for GH-secreting adenomas. First-line drug treatment, with or without primary surgical debulking, is suggested for patients with a low probability of surgical cure [10, 11]. Preoperative growth hormone lowering treatment might improve surgical cure rate in newly diagnosed patients with macroadenomas [25]. Together with improvements in radiotherapeutic techniques, these developments have led to the idea of refraining from surgery in the cavernous sinus. However, alternative surgical techniques, including new (endoscopic) approaches to the cavernous sinus, have demonstrated positive results with low complication rates [26], resulting in pleas for re-evaluation of the role of surgery in the multidisciplinary approach to these invasive adenomas. The endoscopic approach allows for a more extended surgical strategy: removing the bone over the cavernous sinus gives access to its medial wall. Endoscopic intrasellar inspection makes resection of a tumor in the cavernous sinus possible. Surgical control of GH-secreting adenomas is likely better using an extended endoscopic approach [27, 28].

This study accurately analysed patient and tumor characteristics (based on standardised revision of pituitary imaging) influencing outcome of pituitary surgery for GH-secreting adenomas. Nonetheless, some limitations of this study have to be addressed. In our retrospective study the definition of cure is based on the judgement of the attending endocrinologist at the time of treatment. We could not verify in all patients if the biochemical guidelines at time (nadir GH < 1 μg/L and normal IGF-I) [29] were stringently followed. In these cases (n = 2) we based our conclusions on the expert opinion of the endocrinologist, later laboratory results and clinical activity of the disease during follow-up. We believe our study represents clinical practice, but a prospective study using the recent updated consensus on criteria of cure of acromegaly [30] would be more accurate. Thereafter, our prediction model generated from the various patient and tumor characteristics might not be representative for general practice, since the study cohort was relatively small and selection bias could not be avoided completely due to the retrospective nature of the study. Therefore, the results should be interpreted with caution. Models based on larger sample sizes and accurately evaluated characteristics, which are internally and externally validated, need to be generated to provide standardised and valid clinical recommendations. A specific tumor staging classification system is desirable, but even more; randomised studies to compare the effect of different first-line treatment modalities (e.g. medical therapy vs. extended endoscopic surgery) on disease outcome in GH-secreting adenomas with respect to different tumor sizes and invasiveness.

In conclusion, next to male sex, standardised revision of preoperative pituitary MRI scans demonstrated tumor size above 20 mm, infrasellar extension and probable extension into the cavernous sinus tending to be independent predictors of persistent disease after initial surgery. For re-evaluation of the role of surgery in invasive GH-secreting adenomas the need for a practical radiologic classification system for invasiveness is emphasized, and even more; comparative studies on different first-line treatment options, because of the rising availability of these on one hand, and on the other hand because of the evolving surgical techniques.
